# Supplementary material for: Task-related changes in aperiodic activity are related to visual working memory capacity independent of event-related potentials and alpha oscillations
Source: Imaging Neurosci (Camb). 2025 Sep 19;3:IMAG.a.150. doi: 10.1162/IMAG.a.150 (PMC12451300; doi:10.1162/IMAG.a.150)
Supplement: Supplementary Material [file IMAG.a.150_supp.pdf]

**Supplementary material**

# Task-related changes in aperiodic activity are related to visual working memory capacity independent of event-related potentials and alpha oscillations

Sian Virtue-Griffiths [a], Alex Fornito [a], Sarah Thompson [a,b], Mana Biabani [a], Jeggan Tiego [a], Tribikram Thapa [a], Neil W. Bailey [c,d], Nigel C. Rogasch [a,e,f]

a) School of Psychological Sciences, Turner Institute of Brain and Mental Health, and Monash Biomedical Imaging, Monash University, VIC, Australia

b) Clinical Sciences, Murdoch Children's Research Institute, Melbourne, VIC, Australia

c) School of Medicine and Psychology, The Australian National University, Canberra, ACT.

d) Monarch Research Institute, Monarch Mental Health Group, Sydney, NSW.

e) School of Biomedicine, University of Adelaide, SA, Australia

f) Hopwood Centre for Neurobiology, Lifelong Health Theme, South Australian Health and Medical Research Institute, SA, Australia

*Correspondence*

**Sian Virtue-Griffiths**

Email: sian.virtue-griffiths@monash.edu

**Nigel C. Rogasch**

Email: Nigel.rogasch@adelaide.edu.au

*Address:*

Monash University, Building 220, Clayton Campus,

770 Blackburn Rd. Clayton, VIC, 3800, Australia

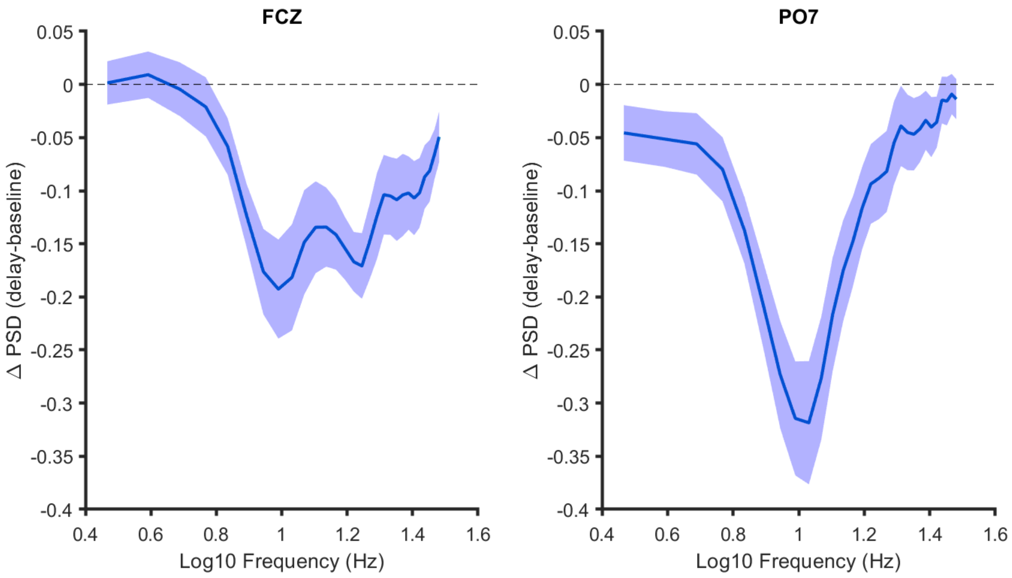


**Supplementary Figure 1.** Differences in the electroencephalography (EEG) power spectral density (PSD) between the delay and baseline periods ($\log_{10}$(delay PSD) – $\log_{10}$(baseline PSD)) for the load 4 condition. The same channels are used as in figure 4 (FCz – frontocentral; and PO7 - occipitoparietal). The solid lines represent the mean difference between the delay and baseline periods and shaded bars represent the 95% confidence intervals. The reduction in power during the delay period compared to the baseline period was less than 0 between 6.8-30.2 Hz in the frontocentral channel, and between 2.9-26.4 Hz in the occipitoparietal channel.

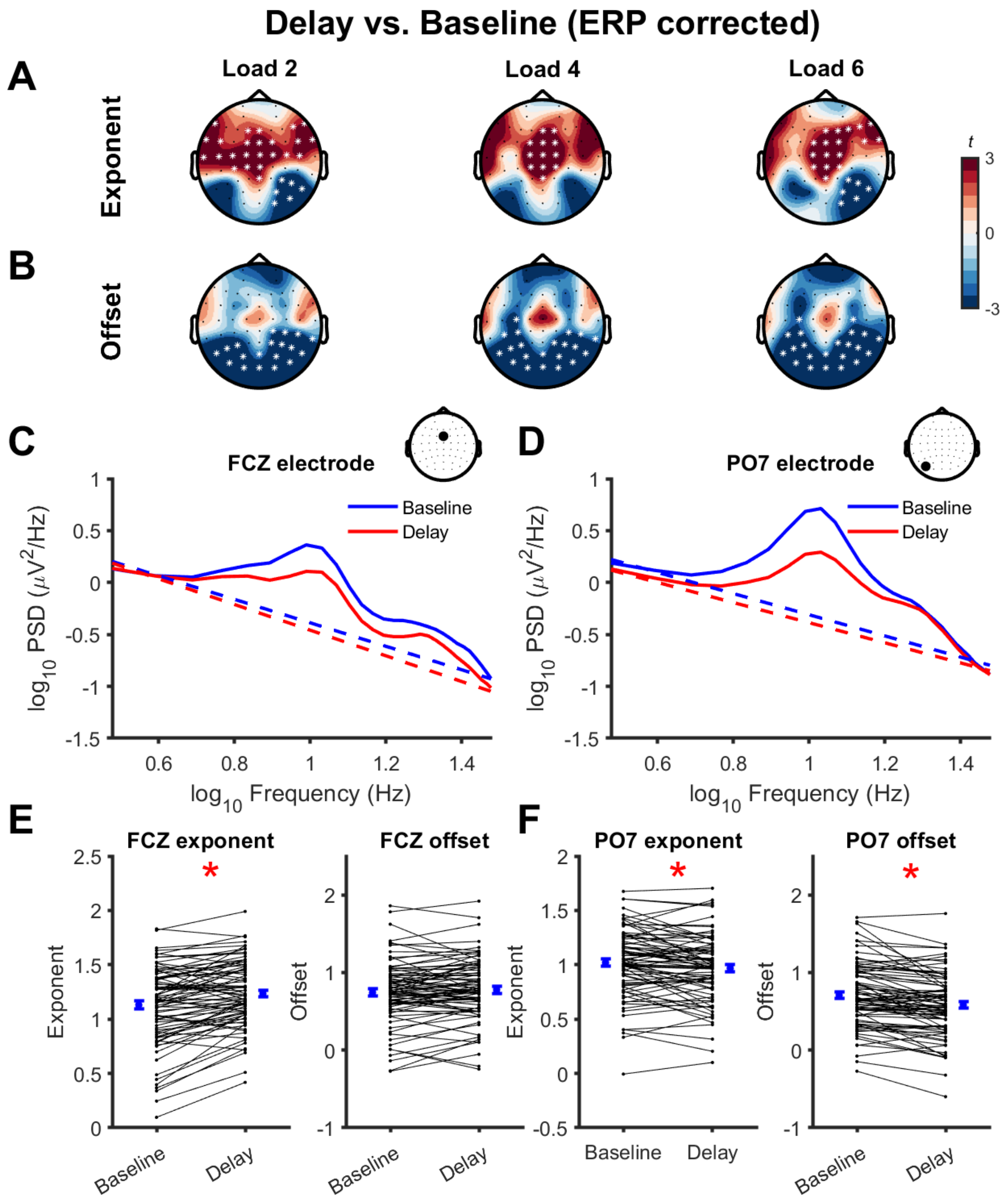


**Supplementary Figure 2.** Task-related changes in aperiodic activity across the scalp after removal of residual ERP activity. Power spectral density (PSD) and aperiodic analyses were conducted after subtracting the ERP from the single trial data in the time domain. A-B) Topoplots representing changes (t-statistics) in aperiodic exponent (A) and offset (B) values between the baseline and delay periods of the working memory task at each load. * indicate electrodes contributing to significant clusters ($p<0.01$). C-D) Group mean EEG power spectra (solid lines) and aperiodic activity (dashed lines) from a frontocentral (C; FCZ) and lateral occipitoparietal (D; PO7) electrode during the baseline (blue) and delay (red) periods of load 4. Data are plotted in log-log space. Inset topoplots indicate the position of the electrode on the scalp. E-F) Group mean (blue dots; ± standard error) and individual (black dots and lines) changes in aperiodic exponent and offset values from baseline to delay periods for frontocentral (E; FCZ) and lateral occipitoparietal (F; PO7) electrodes. * indicate significant differences between time periods ($p<0.05$; paired t-test).
